# Advances in next-generation sequencing for relapsed pediatric acute lymphoblastic leukemia: current insights and future directions

**DOI:** 10.3389/fgene.2024.1394523

**Published:** 2024-06-04

**Authors:** Nur Farhana Mohd Nippah, Nadiah Abu, Nurul Syakima Ab Mutalib, Hamidah Alias

**Affiliations:** ^1^ Department of Pediatrics, Faculty of Medicine, National University of Malaysia, Kuala Lumpur, Malaysia; ^2^ UKM Medical Molecular Biology Institute (UMBI), National University of Malaysia, Kuala Lumpur, Malaysia

**Keywords:** next-generation sequencing, relapsed acute lymphoblastic leukemia, molecular landscape, precision medicine, cancer genome

## Abstract

Leukemia is one of the most common cancers in children; and its genetic diversity in the landscape of acute lymphoblastic leukemia (ALL) is important for diagnosis, risk assessment, and therapeutic approaches. Relapsed ALL remains the leading cause of cancer deaths among children. Almost 20% of children who are treated for ALL and achieve complete remission experience disease recurrence. Relapsed ALL has a poor prognosis, and relapses are more likely to have mutations that affect signaling pathways, chromatin patterning, tumor suppression, and nucleoside metabolism. The identification of ALL subtypes has been based on genomic alterations for several decades, using the molecular landscape at relapse and its clinical significance. Next-generation sequencing (NGS), also known as massive parallel sequencing, is a high-throughput, quick, accurate, and sensitive method to examine the molecular landscape of cancer. This has undoubtedly transformed the study of relapsed ALL. The implementation of NGS has improved ALL genomic analysis, resulting in the recent identification of various novel molecular entities and a deeper understanding of existing ones. Thus, this review aimed to consolidate and critically evaluate the most current information on relapsed pediatric ALL provided by NGS technology. In this phase of targeted therapy and personalized medicine, identifying the capabilities, benefits, and drawbacks of NGS will be essential for healthcare professionals and researchers offering genome-driven care. This would contribute to precision medicine to treat these patients and help improve their overall survival and quality of life.

## 1 Overview of relapsed acute lymphoblastic leukemia (ALL)

ALL is the most prevalent hematological malignancy in children (Roy et al., 2005; Reismüller et al., 2009). The cell/disease arises from the clonal proliferation of lymphoid stem or progenitor cells that have been halted in their maturation, with more than 80% of these cells coming from B-cell progenitors (Hunger and Mullighan, 2015). ALL is characterized by recurrent structural chromosomal changes. Many molecular markers have been found to stratify risk and determine prognosis as cytogenetic changes or molecular abnormalities are relatively common and play pivotal roles in ALL progression (Zhang et al., 2020).

Patients with ALL are divided into standard- and high-risk groups. Risk stratification facilitates the selection of treatment regimens; however, high-risk patients continue to achieve worse outcomes despite receiving more intense therapy (Bohannan et al., 2022). B-precursor leukemia is highly prevalent in children aged between 2 and 5 years, whereas T-ALL most likely occurs in older children at 9 years old (Chan, 2002; Malard and Mohamad, 2020). Younger children perform more effectively in terms of treatment response than older pediatric patients. In contrast, adult patients with ALL perform much worse than children and have a poor prognosis.

With more than 90% long-term survival in high-income countries, the latest advancements in pediatric ALL treatment have significantly improved outcomes (Hunger et al., 2012; Jeha et al., 2019). A combination of drugs and distinct mechanisms of action is needed for effective intense chemotherapy (Malard and Mohamad, 2020), accompanied by thorough outpatient post-remission therapy and followed by continuous low-intensity maintenance chemotherapy to avoid recurrence (Oshima et al., 2020). Nevertheless, patients who experience leukemia relapse often have poor clinical outcomes due to treatment resistance (Ching-Hon Pui and William, 2006; Bhojwani and Pui, 2013).

Next-generation sequencing (NGS) has been recently used to perform genomic profiling of various pediatric ALL subtypes (Mullighan et al., 2007; Holmfeldt et al., 2013; Lindqvist et al., 2015; Ma et al., 2015; Paulsson et al., 2015). Multiple germline genetic variations and somatic changes have been discovered in newly diagnosed and relapsed pediatric ALL or in particular subtypes, which possibly have prognostic consequences (Lindqvist et al., 2015; Paulsson et al., 2015). The characterization of molecular landscapes will provide information on tumor categorization, enabling the development of more efficient treatment regimens and the improvement of patient survival rates. Thus, NGS would be a useful tool for investigating the molecular landscape of relapsed ALL that can lead to therapy-related insights.

## 2 Overview of next-generation sequencing technology

It is important to note that NGS is a revolutionary sequencing technology that has superseded Sanger sequencing, which was initially described in 1977 (Sanger et al., 1977). Technical developments in these sequencing techniques have automated the processes and increased the capacity of sequencing to several thousand base pairs in a single run by substituting fluorescent dyes with radioactive dyes and gel electrophoresis with capillary array electrophoresis (Lohmann and Klein, 2014). These features enabled the use of NGS approaches in various fields, including whole-genome sequencing (WGS), whole-exome sequencing (WES/ES), variant calling (VC), targeted sequencing (TS), and transcriptome sequencing or RNA-seq (Rabbani et al., 2012; Xuan et al., 2013).

WGS involves an examination of the whole nucleotide sequence of a genome (Gkazi, 2021). It has been used in cases where genotype comparison and comprehensive analysis of the genome are necessary, such as when investigating rare disorders (DeWitt, 2020). WGS provides a detailed overview of the cancer genome, which includes analysis of the noncoding regions and the types of somatic and germline mutations, nucleotide substitutions, small insertions, and deletions, copy number variations (CNVs), and chromosomal rearrangements (Meyerson et al., 2010). WGS enables a more detailed analysis that can provide a fuller picture despite the higher, though constantly declining, costs and the associated challenges in data analysis (Gkazi, 2021).

WES is a method of TS that only focuses on the protein-coding exons of the genome (Rabbani et al., 2014). This allows for more specific investigation in these areas as they only make up about 2% of the human genome (Gkazi, 2021). For the analysis of protein-coding genes in a genome, WES is a quick and affordable method frequently used for tumor-normal sequencing (DeWitt, 2020). WES focuses on the roughly 30 million base pairs translated into functional proteins, where mutations are often expected to exert a severe direct phenotypic effect (Bertier et al., 2016). WES can be a more affordable alternative to WGS and minimize the volume and complexity of the sequencing data produced as a result of the reduced sequencing burden. However, merely sequencing a portion of the genome, can cause important information to be overlooked and limit the chance of discoveries (Gkazi, 2021).

WES and WGS are useful approaches in genomic studies, but each has advantages and limitations (Atha, 2023). WES is a practical method for studying exonic regions, as the exome contains more than 80% of disease-causing mutations, even though it is only a relatively small section of the genome (Antonarakis et al., 1995; Choi et al., 2009). By focusing on the exome, WES produces considerably fewer datasets than WGS because less sequencing is required, making the data analysis process simpler and reducing the costs associated with data storage and sequencing. However, one significant drawback of WES is that it cannot cover most of the genome and some exome, which means that significant variations may go unnoticed (Atha, 2023). In contrast, WGS covers the entire genome, but this comprehensive coverage increases the processing costs and may outweigh the potential cost reductions from WES’s partial genome coverage. Moreover, the use of WES limits the ability of researchers to reanalyze data retrospectively because notable variations in non-coding regions can be detected over time (Atha, 2023).

WGS is a more robust and complete technique since it includes data from both coding and non-coding areas. Variants in non-coding areas can impact genes that would not be detected in WES in terms of expression or splicing. The benefits of long-read sequencers in WGS underscore its capacity to provide supplementary regulatory data, such as 5mC, that is not available in WES (Atha, 2023). Compared to WES, which occasionally suffers from unequal probe coverage, WGS provides more reliable and precise coverage of exonic regions. WGS is more efficient and involves fewer steps in the wet lab workflow, which may make it better suited for validation in subsequent clinical contexts. Notwithstanding these many benefits, WGS is more expensive than WES because it demands more sequencing. Additionally, it generates a significantly higher number of data, necessitating the use of specialist bioinformatics tools. Consequently, compared to WES, the analysis process is substantially longer and needs more storage (Atha, 2023).

In addition, the emergence of NGS has revolutionized studies on cancer transcriptomics (Nones and Patch, 2020). RNA sequencing (RNA-seq) is a promising NGS tool that can concurrently detect cryptic gene rearrangements, sequence alterations, and gene expression profiles. Although all these findings can be detected from bulk RNA-seq, various bioinformatics algorithms must be used to detect each one. RNA-seq has gradually become one of the most effective techniques for genome-wide expression profiling. It identifies some genetic changes that can potentially exert prognostic and therapeutic effects but can be overlooked with more conventional approaches (Tran et al., 2022).

NGS has been used in ALL research for more than a decade. It has facilitated and improved the identification of important molecular abnormalities (Suhaimi et al., 2016). Therefore, we reviewed the most recent publications in this field (Table 1).

**TABLE 1:** Latest major research on the use of next-generation sequencing (NGS) in relapsed acute lymphoblastic leukemia (ALL).

No.	Authors	Samples analyzed	Type of NGS approach	Main findings
1	Vesely et al. (2017)	DNA or frozen viable cells from 41 significant clone fusion-positive BCP-ALL patients with 19 matched diagnosis/relapse pairings for WES	WES, RNA-seq	• In 76% of cases at diagnosis and nearly all relapses, a range of frequently subclonal, extremely unstable, and *JAK/STAT* as well as *RTK/Ras* pathway-activating mutations were found
				• *IKZF1* alterations increased from 36% to 58% in matched patients and were more prevalent in relapsed cases (*p* = 0.001)
2	Ding et al. (2017)	DNA samples from 240 pediatric ALL patients with their matched remission samples	WES, TS	• The *RAS*/receptor tyrosine kinases, epigenetic regulators, transcriptional factors involved in lineage commitment, and *p53*/cell cycle pathway were among the groups of genes that were often altered
				• The tyrosine kinase *FLT3* (*K663R*, *N676K*) and the epigenetic regulators; *WHSC1(E1099K)* and *CREBBP* (*R1446C/H*) were shown to have specific recurring mutational hotspots
				• The epigenetic regulator *ARID1A* and transcriptional factor *CTCF* were effectively found as potential tumor suppressors, whereas the mutant *WHSC1* was identified as a gain-of-function oncogene
3	Kimura et al. (2019)	30 cases of matched T-ALL and normal (samples in the complete-remission phase) pairs, including 11 trios comprising samples at relapse, samples at diagnosis, and normal samples	WES	• *NOTCH1/FBXW7* abnormalities were found in 73.3% (diagnosis) and 72.7% (relapse) of patients
				• PEST alterations were more prominent in patients with relapse (*p* = .045) than in nonrelapse patients at diagnosis
				• In 2 out of 11 diagnosis–relapse paired cases studied, *NOTCH1* “switching” was found, which is affected by unique *NOTCH1* mutations in the main clone between diagnostic and relapse samples
4	Zhang et al. (2020)	BM samples were obtained at the time of diagnosis and matched with remission samples from 140 Chinese pediatric ALL patients	Targeted exome sequencing	• B-ALL patients showed the most mutated genes of *KRAS*, *NRAS,* and *FLT3* whereas T-ALL patients enriched with *NOTCH1*, *FBXW7*, and *PHF6* mutations
				• Among 18 altered genes, *SETD2* and *TP53* mutations were more common in female patients (*p* = 0.041), *NOTCH1* and *SETD2* mutations had higher initial WBC counts (*p* = 0.041), and *JAK1* mutations had higher (MRD) levels after induction chemotherapy
				• Initial WBC counts, *MLLr*, and *TP53* mutations were identified as independent risk factors for 3-year relapse-free survival (RFS) in ALL via multivariate analysis
5	Hoell et al. (2019)	Leukemic blasts (DNA) from 10 children with post-allo-SCT relapses	WES	• Genetic lesions in post-allo-SCT ALL relapses are quite varied and typically patient-specific
				• Mutational cluster analysis showed significant clonal dynamics throughout leukemia, from the initial diagnosis to relapse post-allo-SCT
				• Detected *TP53* mutations in 4 of 10 patients post-allo-SCT
				• Genetic alterations were detected in 9 out of 10 children with post-allo-SCT relapse
6	Sentís et al. (2020)	Samples were obtained at the time of diagnosis and relapse from 19 adult patients with T-ALL	WGS	• Before the primary T-ALL is diagnosed, the relapse clone first appears
				• In at least 14 of the 19 patients, the population of relapse leukemia established at the time of diagnosis contained more than 1 but fewer than 108 blasts through the doubling time of the leukemic population
7	Waanders et al. (2020)	Tumor samples from 92 cases of relapsed pediatric ALL	WGS, WES, RNA-seq	• 50 Major mutational targets with unique mutational acquisition or enrichment patterns have been found
				• *CREBBP*, *NOTCH1*, and *RAS* signaling mutations developed from diagnostic subclones, whereas *NCOR2*, *USH2A*, and *NT5C2* variations were only detected during relapse
8	Li et al. (2020)	103 Diagnosis–relapse–germline trios and ultra-deep sequencing of 208 serial samples in 16 patients	WES	• 12 Genes associated with drug response were enriched for relapse-specific somatic changes
				• Two unique relapse-specific mutational signatures were observed in early and late relapses, which were attributed to thiopurine treatment
				• *NT5C2*, *PRPS1*, *NR3C1,* and *TP53* acquired resistance mutations accounted for 46% of the new signatures observed in 27% of relapsed ALLs
9	Yu et al. (2020)	BM samples from eight matched diagnosis–remission–relapse triplicate ALL samples	WES	• Relapse-specific mutations in the gene for *FPGS* were observed in one patient
				• Six patients had *NT5C2* mutations, two had *PRPS1* mutations, and two had three additional *FPGS* mutations
				• One patient had both *NT5C2* and *PRPS1* mutations
10	Oshima et al. (2020)	DNA and leukemia lymphoblast samples from 175 ALL patients were obtained at diagnosis, at remission, and after relapse	WGS, WES, RNA-seq	• *JAK1* and *JAK3* mutations were found to be co-occurring in T-ALL at diagnosis and also *JAK1* and *WHSC1* mutations after relapse
				• *SETD2* mutations and *ETV6* deletions, as well as *NRAS* and *CREBBP* mutations upon relapse, were all significantly associated with one another in B-precursor ALL
				• The well-known oncogenes and tumor suppressors with recurring somatic mutations detected at diagnosis were *KRAS*, *NRAS*, and *PTPN11* in B-cell precursor ALL and *NOTCH1*, *FBXW7,* and *MYC* in T-ALL
				• Recurrent somatic *TP53*, *NT5C2*, and *CREBBP* mutations that occur mostly or exclusively during relapse
11	Sayyab et al. (2021)	BM or blood samples were collected at diagnosis, remission, and relapse from 29 patients with ALL and were analyzed, including 2 consecutive relapse samples from 9 patients	WGS, RNA-seq	• A higher burden of somatic mutations was observed at relapse than at diagnosis and at the second relapse than at the first relapse
				• Discovered probable nonprotein-coding mutations in the regulatory domains of an additional seven genes, in addition to the 29 known ALL-driver genes, 9 of which had recurring protein-coding mutations in the sample set
				• Three unique evolutionary paths were found throughout the ALL progression from diagnosis to relapse via cluster analysis of hundreds of somatic mutations per sample
12	Antić et al. (2022)	Samples from diagnosis, complete-remission, and relapse from 12 pediatric BCP-ALL patients who experienced very early BM relapse	WGS, WES	• Detected an active clonal evolution in every case, with relapse virtually always arising from a subclone at diagnosis
				• Found several driver mutations that might have affected a minor clone at diagnosis to develop into a significant clone at relapse
				• The *E1099K WHSC1* mutation, a hotspot mutation repeatedly observed in other very early *TCF3–PBX1*-positive leukemia relapses, was present in two patients with *TCF3–PBX1*-positive leukemia who experienced a very early recurrence
13	Shirai et al. (2022)	DNA was extracted from tumor specimens at diagnosis and paired blood samples at remission for six patients, and tumor-only analysis was conducted for one patient whose remission sample was unavailable	WES	• Frequent alterations included 6q *LOH* and *KMT2D* variants
				• Out of the seven patients, 6q *LOH* was found in two
				• The two patients had a 6q deletion region that spanned 6q12–6q16.3
14	Zou et al. (2022)	About 283 patients were enrolled in the study. BM samples were obtained from 25 patients at diagnosis and/or on relapse for WES, and RNA-seq was performed for 6 patients at relapse	WES, RNA-seq	• Two patients had detectable germline mutations, *TP53* or *FLT3*, 14 or 15 patients had somatic mutations identified at diagnosis or relapse, and five patients had unfavorable mutations, *TP53*, *CREBBP*, and *IKZF1*
				• Six patients had molecular abnormalities, and three had reversed molecular abnormalities (*CREBBP*, *TP53*, and *P2RY-CRLF2*); other unreported genetic abnormalities in B-ALL, including *TPM4-KLF2* or *NR3C1-CDC42* transcript, were also discovered

^a^ Whole-genome sequencing (WGS); transcriptome sequencing (RNA-seq); whole-exome sequencing (WES); B-cell precursor acute lymphoblastic leukemia (BCP-ALL); allogeneic stem cell transplantation (allo-SCT); bone marrow (BM).

### 2.1 WGS reveals genetic events that contribute to disease development

In 2020, both Li et al. (2020) and Sentís et al. (2020) performed WGS which both focused on the somatic alterations observed during diagnosis and relapsed stage for both B-ALL and T-ALL patients. SNVs, indels, copy number variations (CNVs), and structural variations (SVs) were identified. Li et al. (2020) reported that 12 genes were enriched in relapse-specific alterations, including 11 known relapse-related genes. Seven of the 12 genes showed relapse-specific alterations in both B-ALL and T-ALL, whereas *PRPS1, MSH2, FPGS, CREBBP*, and *WHSC1* alterations were exclusively observed in B-ALL. Sentís et al. (2020) compared the mutational profiles of T-ALL and B-ALL patients of varying ages and found no significant difference. However, slight variations were observed between pediatric and adult malignancies. The study also revealed that *NOTCH1* and *FBXW7* were overrepresented in both pediatric and adult T-ALL as compared to B-ALL (Sentís et al., 2020).

Waanders studied the genomic patterns and mutational pathways that cause relapse in pediatric patients with relapsed B-ALL and T-ALL (Waanders et al., 2020). Fifty genes were enriched as mutational targets in relapsed diseases. Epigenetic regulators such as *PRDM2, PHF19, TET3,* and *SIN3A,* were also enriched in the relapse samples. Mutations observed in different gene regulation pathways showed different frequencies of relapse-enriched genes between B- and T-ALL, implying that distinct biological mechanisms drive the genetic alterations responsible for disease progression (Waanders et al., 2020). Oshima et al. (2020) analyzed paired diagnostic and relapse samples from children and adults with B precursor and T-ALL. They found an average of 27–34 coding mutations in diagnostic and relapse samples, respectively. Mutational signatures associated with microsatellite instability were observed along with significant associations between certain mutations at diagnosis and relapse. In T-ALL, they observed a significant co-occurrence of *JAK1* and *JAK3* mutations (Oshima et al., 2020), which was similarly reported in a previous study (Sentís et al., 2020), and an association between *WT1* and *NRAS* mutations, and between *JAK1* and *WHSC1* mutations at relapse (Oshima et al., 2020). In B precursor ALL, a significant association was observed between *SETD2* mutations and *ETV6* deletions and between *NRAS* and *CREBBP* mutations at relapse (Oshima et al., 2020).

Antic et al. performed WGS on 10 samples from 2 pediatric BCP-ALL patients with multiple relapses (Antic et al., 2021). They detected 8,922 and 8,759 single-base substitutions and 686 and 646 indels in patients 1 and 2, respectively. Signatures for single-base substitutions (SBS)2 and SBS13 were established at diagnosis and persisted throughout disease progression (Antić et al., 2022). Sayyab et al. (2021) studied eight subtypes of BCP-ALL, the B-other subgroup, and T-ALL. They reported that somatic mutations differed between subtypes and individual samples. Nine ALL-driver genes had recurrent protein-coding mutations. In addition, copy-number aberrations and deletions of 9p21.3 remained high from diagnosis to relapse (Sayyab et al., 2021).

In summary, several investigations utilized WGS and analyzed samples obtained during diagnosis and relapse stages to identify somatic changes in different subtypes of ALL. The results of these studies varied depending on their objectives, with different somatic mutational patterns observed in pediatric and adult patients with ALL based on the specific subtype of the disease.

### 2.2 WES reveals genetic variations associated with relapsed ALL

In 2017, Vesely et al. (2017) analyzed WES data from 41 patients with leukemic BCP-ALL and found subclonal and unstable *JAK/STAT*- and *RTK/Ras* pathway-activating mutations in 76% of cases at diagnosis and almost all relapses. Because the *P2RY8–CRLF2* fusion, a *JAK/STAT-* or *RTK/Ras* pathway mutation, or all three, was lost at relapse (Vesely et al., 2017). Ding et al. (2017) later performed WES and TS in 11 trios of B-ALL patients with diagnoses, full remissions, and relapses. They found the *RTK-Ras* signaling pathway, epigenetic regulators, transcriptional factors, and p53/cell cycle pathway were the most significantly mutated genes throughout the entire cohort (Ding et al., 2017).

Kimura et al. examined 30 cases of T-ALL, encompassing both initial diagnosis and relapse, through WES (Kimura et al., 2019). Their findings indicated a higher mutation rate in relapsed samples than in those at the time of diagnosis. At the outset, *NOTCH1, FBXW7, DNM2,* and *PHF6* mutations were common, with *NOTCH1* mutations continuing to appear frequently during relapse. PEST-rich alterations were more prevalent in relapsed cases than in non-relapsed cases at diagnosis (Kimura et al., 2019). Similarly, Hoell et al. (2019) studied leukemic blasts from 10 children with post-allogeneic stem cell transplantation (SCT) relapses and discovered that *NOTCH1* was the sole recurrent gene among the 50 genes in T-cell leukemia. In their study using targeted exome sequencing, Zhang et al. (2020) observed distinct mutation patterns between B-ALL and T-ALL patients. *KRAS* was most frequently mutated in B-ALL, whereas T-ALL exhibited enrichment for *NOTCH1*, *FBXW7, PHF6,* and *PTEN* mutations (Zhang et al., 2020), which is consistent with the findings of Ding et al. (2017). Both B-ALL and T-ALL frequently show mutations in the *Ras* and *Notch* pathways (Zhang et al., 2020).

In 2020, Yu et al. (2020) employed WES and examined 299 diagnostic and 73 relapse samples from 372 ALL patients to assess the incidence of *FPGS* mutations, as well as those in two crucial thiopurine pathway genes, *NT5C2* and *PRPS1* which have been previously demonstrated to be important mechanisms for resistance in leukemia (Schroeder et al., 2019). Three additional *FPGS* mutations were detected in two patients, *NT5C2* mutations in six, *PRPS1* mutations in two, and both *NT5C2* and *PRPS1* mutations in one. According to Yu et al. (2020), three children with relapsed ALL had four acquired relapse-specific mutations in *FPGS*. Unexpectedly, Schroeder et al. (2019) and Li et al. (2020) identified a new relapse-specific mutation in the *FPGS* gene that solely affected B-ALL and exhibited relapse-specific lesions and stable losses. In addition, similar investigations concluded that most of these changes were induced by a subclone or were acquired during relapse (Schroeder et al., 2019; Li et al., 2020). Therefore, *FPGS* relapse-specific mutations constitute a pharmacogenetic pathway associated with pediatric ALL relapse and should be regarded as a factor of relapse in pediatric ALL (Yu et al., 2020).

In a study conducted by Oshima et al. (2020), WES was performed on matched germline diagnosis and relapse DNA samples. This study revealed diagnostic and relapse-specific mutational mechanisms, along with genetic chemoresistance drivers. Somatic copy number variations (CNVs) were identified, with an average of 18 somatic CNVs per sample for 6,475 alterations in the series. Of these, 3,589 CNVs were detected at the time of diagnosis and 2,876 at the time of relapse, with 2,575 variants present in both the diagnostic and relapsed samples (Oshima et al., 2020).

In 2021, deep sequencing validation of all detected mutations was performed by Antic et al. (2021) after conducting genomic analysis of diagnosis–relapse paired samples of patients who relapsed very early. Two patients with *TCF3–PBX1*-positive leukemia who experienced very early relapse had *E1099K WHSC1* mutations at the time of diagnosis. This hotspot mutation has also been frequently detected in other cases of very early *TCF3–PBX1*-positive leukemia relapses. This finding suggests that minor subclones at diagnosis typically cause early relapses in BCP-ALL (Antic et al., 2021).

Shirai et al. reported that, in this investigation, *KMT2D* mutations and 6q LOH were found to be recurring changes in seven patients with *TCF3–PBX1*-positive B-LBL (Shirai et al., 2022). Additional genetic mutations were found in the relapsed tumor that were not present in the primary tumor, including the 6q LOH. Analysis of recurrent cases revealed that the relapsed clone may have originated from a minor BM clone at the time of diagnosis (Shirai et al., 2022). According to Zou et al. (2022)’s analysis of data from patients with BM relapse, 62.5% of patients with BM relapse had abnormalities discovered using NGS, and uncommon or previously unreported fusion genes and/or gene mutations were detected. Patients with adverse molecular genetic mutations, such as *TP53, CREBBP,* and *IKZF1* had their BMs relapsed (Forero-Castro et al., 2017; Montaño et al., 2020).

**FIGURE 1:**
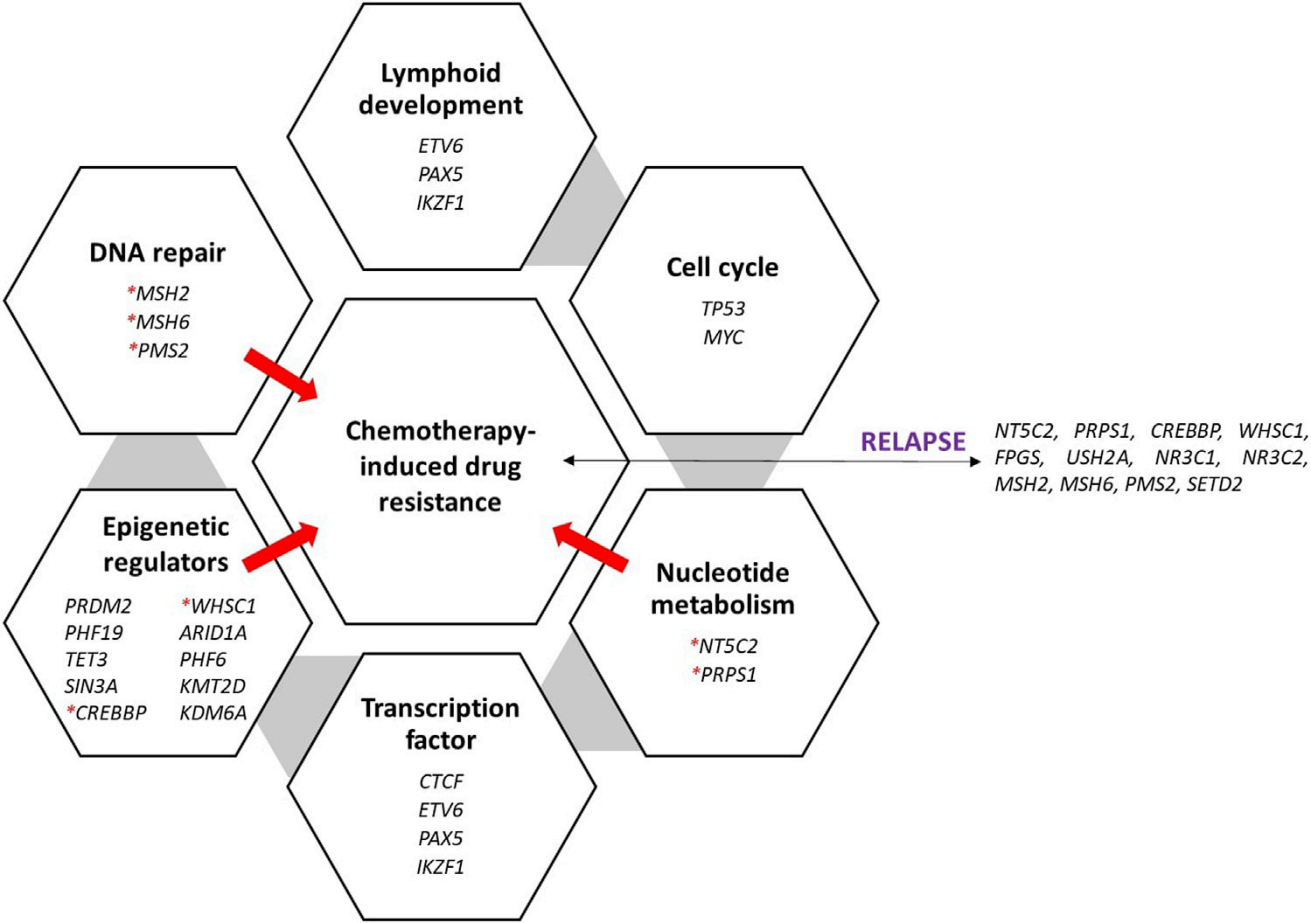
Recurrently mutated genes and pathways in ALL and the various roles of genes that are significantly mutated in ALL. The genes were discovered during relapse and caused treatment resistance. Red asterisks denote the genes that cause the ALL to be resistant to treatment, thus causing relapse.

As an outcome, using all these findings, this pharmacogenetic knowledge and research can be very helpful in situations with limited resources for identifying the requirements of patients based on their genetic risk profiles; this situation is also being resolved. Hence, Figure 1 presents the genes involved in chemotherapy resistance that cause relapse and play various roles.

### 2.3 RNA-seq shows mutational patterns in pediatric relapsed ALL

In 2017, Vesely et al. (2017) performed RNA-seq and discovered that *IKZF1* mutations result in distinct transcriptional signatures. They studied the RNA-seq data based on IKZF1 status, as IKZF1 alterations are strongly associated with relapses in *P2RY8–CRLF2*-positive ALL cases. The 200 genes with the highest differential expression in the IKN and IKD groups were clustered by *IKZF1* status using unsupervised hierarchical clustering. This observation was supported by secondary RNA-seq data collection of 20 B-other ALL cases with known *IKZF1* status (Vesely et al., 2017).

In 2020, Waanders et al. (2020) performed RNA-seq on 115 samples made up of TRIzol-extracted RNA samples from 66 patients. They discovered that the expected human leukocyte antigen (HLA)--binding mutant peptide count per tumor increased with disease progression due to a higher mutation burden, and hence, notably in hypermutated samples (Waanders et al., 2020). With increased neoepitope burden and possible immunotherapy vulnerability, a subset of leukemia predisposed to recurring relapse exhibits hypermutation caused by at least three different mutational mechanisms (Waanders et al., 2020).

Oshima et al. conducted chimeric gene transcript analyses using RNA-seq data for 85 cases (Oshima et al., 2020). These analyses revealed the existence of oncogenic gene transcripts as well as a possible initiating rearrangement between *TBL1XR1* and *JAK2*. Despite the exception of two patients who tested positive only at relapse, one for *PICALM–MLLT10* and the other for *NUP214–ABL*, fusion oncogene transcripts were typically observed at both diagnosis and relapse (Oshima et al., 2020).

In 2021, Sayyab et al. (2021) performed RNA-seq and identified genome-wide patterns of somatic mutations in pediatric Nordic ALL patients. RNA-seq libraries were constructed using the RNA from 22 ALL patients who were diagnosed, 25 patients who experienced their first relapse, and 7 patients who experienced their second relapse, as well as the control B cells (CD19^+^) and T cells (CD3^+^) from five Swedish healthy individuals. RNA-seq data showed evidence of the involvement of exons in gene fusion (Sayyab et al., 2021).

Zou et al. (2022) studied pediatric B-ALL patients without specific fusion genes for outcomes and risk factors in three Chinese institutes. Analysis of the RNA-seq data revealed molecular abnormalities in six patients and reversed molecular abnormalities in three (*CREBBP, TP53,* and *P2RY-CRLF2*), as well as previously unreported genetic mutations in B-ALL (Zou et al., 2022).

In terms of transcriptional characteristics including newly transcribed regions, RNA editing, allele-specific expression, variant splicing, RNA sequencing, or RNA-seq offers a previously unheard-of accuracy. Owing to these advancements, the transcriptome and its implications for fundamental biology and personalized medicine may now be studied using a powerful toolkit.

## 3 Conclusion

The introduction of NGS has generated an effective and widely used approach. Moreover, the assay specificity has increased to unprecedented levels. The application of NGS has improved the understanding of tumor genomic heterogeneity, exhibited significant ramifications, and made clinical decision-making for tailored therapeutic interventions. In this review, we collected and discussed studies on NGS-based relapsed ALL. Owing to its capability to identify significant genome modifications, NGS has transformed both ALL and relapsed ALL genomics. NGS holds great promise for the future, opening up intriguing channels for research and spurring development that will significantly influence the understanding of genome-guided knowledge and personalized medicine.

## References

[B1] Antic Ž. Lelieveld S. H. Van Der Ham C. G. Sonneveld E. Hoogerbrugge P. M. Kuiper R. P. (2021). Unravelling the sequential interplay of mutational mechanisms during clonal evolution in relapsed pediatric acute lymphoblastic leukemia. Genes (Basel) 12 (2), 214. 10.3390/genes12020214 33540666 PMC7913080

[B2] Antić Ž. Yu J. Bornhauser B. C. Lelieveld S. H. van der Ham C. G. van Reijmersdal S. V. (2022). Clonal dynamics in pediatric B-cell precursor acute lymphoblastic leukemia with very early relapse. Pediatr. Blood Cancer 69 (1), e29361. 10.1002/pbc.29361 34597466

[B3] Antonarakis S. E. Krawczak M. Cooper D. N. (1995) The nature and mechanisms of human gene mutation. The online metabolic and molecular bases of inherited disease.

[B4] Atha B. B. (2023) When to use whole-genome vs. Whole-exome sequencing.

[B5] Bertier G. Hétu M. Joly Y. (2016). Unsolved challenges of clinical whole-exome sequencing: a systematic literature review of end-users' views. BMC Med. Genomics 9 (1), 52. 10.1186/s12920-016-0213-6 27514372 PMC4982236

[B6] Bhojwani D. Pui C. H. (2013) Relapsed childhood acute lymphoblastic leukaemia. Vol. 14. Available at: www.thelancet.com/oncology. 10.1016/S1470-2045(12)70580-6 23639321

[B7] Bohannan Z. S. Coffman F. Mitrofanova A. (2022). Random survival forest model identifies novel biomarkers of event-free survival in high-risk pediatric acute lymphoblastic leukemia. Comput. Struct. Biotechnol. J. 20, 583–597. 10.1016/j.csbj.2022.01.003 35116134 PMC8777142

[B8] Chan K. W. (2002) Acute lymphoblastic leukemia. 10.1067/mps.2002.121790 11951089

[B9] Ching-Hon Pui M. D. William E. (2006). Treatment of acute lymphoblastic leukemia. N. Engl. J. Med. 354, 166–178. 10.1056/nejmra052603 16407512

[B10] Choi M. Scholl U. I. Ji W. Liu T. Tikhonova I. R. Zumbo P. (2009). Genetic diagnosis by whole exome capture and massively parallel DNA sequencing. Proc. Natl. Acad. Sci. U. S. A. 106, 19096–19101. 10.1073/pnas.0910672106 19861545 PMC2768590

[B11] DeWitt J. (2020). “Integrated DNA technologies,” in Beginner’s guide to next generation sequencing.

[B12] Ding L. W. Sun Q. Y. Tan K. T. Chien W. Thippeswamy A. M. Yeoh A. E. J. (2017). Mutational landscape of pediatric acute lymphoblastic leukemia. Cancer Res. 77 (2), 390–400. 10.1158/0008-5472.CAN-16-1303 27872090 PMC5243866

[B13] Forero-Castro M. Robledo C. Benito R. Bodega-Mayor I. Rapado I. Hernández-Sánchez M. (2017). Mutations in TP53 and JAK2 are independent prognostic biomarkers in B-cell precursor acute lymphoblastic leukaemia. Br. J. Cancer 117 (2), 256–265. 10.1038/bjc.2017.152 28557976 PMC5520505

[B14] Gkazi A. (2021) An overview of next-generation sequencing.

[B15] Hoell J. I. Ginzel S. Kuhlen M. Kloetgen A. Gombert M. Fischer U. (2019). Pediatric ALL relapses after allo-SCT show high individuality, clonal dynamics, selective pressure, and druggable targets. Blood Adv. 3 (20), 3143–3156. 10.1182/bloodadvances.2019000051 31648313 PMC6849953

[B16] Holmfeldt L. Wei L. Diaz-Flores E. Walsh M. Zhang J. Ding L. (2013). The genomic landscape of hypodiploid acute lymphoblastic leukemia. Nat. Genet. 45 (3), 242–252. 10.1038/ng.2532 23334668 PMC3919793

[B17] Hunger S. P. Lu X. Devidas M. Camitta B. M. Gaynon P. S. Winick N. J. (2012). Improved survival for children and adolescents with acute lymphoblastic leukemia between 1990 and 2005: a report from the children’s oncology group. J. Clin. Oncol. 30 (14), 1663–1669. 10.1200/JCO.2011.37.8018 22412151 PMC3383113

[B18] Hunger S. P. Mullighan C. G. (2015). Acute lymphoblastic leukemia in children. N. Engl. J. Med. 373, 1541–1552. 10.1056/NEJMra1400972 26465987

[B19] Jeha S. Pei D. Choi J. Cheng C. Sandlund J. T. Coustan-Smith E. (2019). Improved CNS control of childhood acute lymphoblastic leukemia without cranial irradiation: st jude total therapy study 16. J. Clin. Oncol. 37, 3377–3391. 10.1200/JCO.19.01692 31657981 PMC7351342

[B20] Kimura S. Seki M. Yoshida K. Shiraishi Y. Akiyama M. Koh K. (2019). NOTCH1 pathway activating mutations and clonal evolution in pediatric T-cell acute lymphoblastic leukemia. Cancer Sci. 110 (2), 784–794. 10.1111/cas.13859 30387229 PMC6361559

[B21] Li B. Brady S. W. Ma X. Shen S. Zhang Y. Li Y. (2020). Therapy-induced mutations drive the genomic landscape of relapsed acute lymphoblastic leukemia. Blood 135 (1), 41–55. 10.1182/blood.2019002220 31697823 PMC6940198

[B22] Lindqvist C. M. Nordlund J. Ekman D. Johansson A. Moghadam B. T. Raine A. (2015). The mutational landscape in pediatric acute lymphoblastic leukemia deciphered by whole genome sequencing. Hum. Mutat. 36 (1), 118–128. 10.1002/humu.22719 25355294 PMC4309499

[B23] Lohmann K. Klein C. (2014). Next generation sequencing and the future of genetic diagnosis. Neurotherapeutics 11, 699–707. Springer Science and Business Media, LLC. 10.1007/s13311-014-0288-8 25052068 PMC4391380

[B24] Ma X. Edmonson M. Yergeau D. Muzny D. M. Hampton O. A. Rusch M. (2015). Rise and fall of subclones from diagnosis to relapse in pediatric B-acute lymphoblastic leukaemia. Nat. Commun. 6, 6604. 10.1038/ncomms7604 25790293 PMC4377644

[B25] Malard F. Mohamad M. (2020) Acute lymphoblastic leukaemia. Available at: www.thelancet.com. 10.1016/S0140-6736(19)33018-1 32247396

[B26] Meyerson M. Gabriel S. Getz G. (2010). Advances in understanding cancer genomes through second-generation sequencing. Nat. Rev. Genet. 11, 685–696. 10.1038/nrg2841 20847746

[B27] Montaño A. Hernández-Sánchez J. Forero-Castro M. Matorra-Miguel M. Lumbreras E. Miguel C. (2020). Comprehensive custom ngs panel validation for the improvement of the stratification of b-acute lymphoblastic leukemia patients. J. Pers. Med. 10 (3), 137. 10.3390/jpm10030137 32967112 PMC7565730

[B28] Mullighan C. G. Goorha S. Radtke I. Miller C. B. Coustan-Smith E. Dalton J. D. (2007). Genome-wide analysis of genetic alterations in acute lymphoblastic leukaemia. Nature 446, 758–764. Nature Publishing Group. 10.1038/nature05690 17344859

[B29] Nones K. Patch A. M. (2020). The impact of next generation sequencing in cancer research. Cancers 12, 29288–E2934. MDPI AG. 10.3390/cancers12102928 PMC7601779 33053644

[B30] Oshima K. Zhao J. Pérez-Durán P. Brown J. A. Patiño-Galindo J. A. Chu T. (2020). Mutational and functional genetics mapping of chemotherapy resistance mechanisms in relapsed acute lymphoblastic leukemia. Nat. Cancer 1 (11), 1113–1127. 10.1038/s43018-020-00124-1 33796864 PMC8011577

[B31] Paulsson K. Lilljebjörn H. Biloglav A. Olsson L. Rissler M. Castor A. (2015). The genomic landscape of high hyperdiploid childhood acute lymphoblastic leukemia. Nat. Genet. 47 (6), 672–676. 10.1038/ng.3301 25961940

[B32] Rabbani B. Mahdieh N. Hosomichi K. Nakaoka H. Inoue I. (2012). Next-generation sequencing: impact of exome sequencing in characterizing Mendelian disorders. J. Hum. Genet. 57, 621–632. 10.1038/jhg.2012.91 22832387

[B33] Rabbani B. Tekin M. Mahdieh N. (2014). The promise of whole-exome sequencing in medical genetics. J. Hum. Genet. 59, 5–15. 10.1038/jhg.2013.114 24196381

[B34] Reismüller B. Attarbaschi A. Peters C. Dworzak M. N. Pötschger U. Urban C. (2009). Long-term outcome of initially homogenously treated and relapsed childhood acute lymphoblastic leukaemia in Austria - a population-based report of the Austrian Berlin-Frankfurt-Münster (BFM) Study Group. Br. J. Haematol. 144 (4), 559–570. 10.1111/j.1365-2141.2008.07499.x 19077160

[B35] Roy A. Cargill A. Love S. Moorman A. V. Stoneham S. Lim A. (2005). Outcome after first relapse in childhood acute lymphoblastic leukaemia - lessons from the United Kingdom R2 trial. Br. J. Haematol. 130 (1), 67–75. 10.1111/j.1365-2141.2005.05572.x 15982346

[B36] Sanger F. Nicklen S. Coulson A. R. (1977) DNA sequencing with chain-terminating inhibitors (DNA polymerase/nucleotide sequences/bacteriophage 4X174). Vol. 74. Available at: https://www.pnas.org.

[B37] Sayyab S. Lundmark A. Larsson M. Ringnér M. Nystedt S. Marincevic-Zuniga Y. (2021). Mutational patterns and clonal evolution from diagnosis to relapse in pediatric acute lymphoblastic leukemia. Sci. Rep. 11 (1), 15988. 10.1038/s41598-021-95109-0 34362951 PMC8346595

[B38] Schroeder M. P. Bastian L. Eckert C. Gökbuget N. James A. R. Tanchez J. O. (2019). Integrated analysis of relapsed B-cell precursor Acute Lymphoblastic Leukemia identifies subtype-specific cytokine and metabolic signatures. Sci. Rep. 9 (1), 4188. 10.1038/s41598-019-40786-1 30862934 PMC6414622

[B39] Sentís I. Gonzalez S. Genescà E. García-Hernández V. Muiños F. Gonzalez C. (2020). The evolution of relapse of adult T cell acute lymphoblastic leukemia. Genome Biol. 21 (1), 284. 10.1186/s13059-020-02192-z 33225950 PMC7682094

[B40] Shirai R. Osumi T. Sato-Otsubo A. Nakabayashi K. Mori T. Yoshida M. (2022). Genetic features of B-cell lymphoblastic lymphoma with TCF3-PBX1. Cancer Rep. 5 (9), e1559. 10.1002/cnr2.1559 PMC9458492 34553842

[B41] Suhaimi S. S. Ab Mutalib N. S. Jamal R. (2016). Understanding molecular landscape of endometrial cancer through next generation sequencing: what we have learned so far? Front. Pharmacol. 7, 409. Frontiers Media S.A. 10.3389/fphar.2016.00409 27847479 PMC5088199

[B42] Tran T. H. Langlois S. Meloche C. Caron M. Saint-Onge P. Rouette A. (2022). Whole-transcriptome analysis in acute lymphoblastic leukemia: a report from the DFCI ALL Consortium Protocol 16-001. Blood Adv. 6 (4), 1329–1341. 10.1182/bloodadvances.2021005634 34933343 PMC8864659

[B43] Vesely C. Frech C. Eckert C. Cario G. Mecklenbräuker A. Zur Stadt U. (2017). Genomic and transcriptional landscape of P2RY8-CRLF2-positive childhood acute lymphoblastic leukemia. Leukemia 31 (7), 1491–1501. 10.1038/leu.2016.365 27899802 PMC5508072

[B44] Waanders E. Gu Z. Dobson S. M. Antić Ž. Crawford J. C. Ma X. (2020). Mutational landscape and patterns of clonal evolution in relapsed pediatric acute lymphoblastic leukemia. Blood Cancer Discov. 1 (1), 96–111. 10.1158/0008-5472.BCD-19-0041 32793890 PMC7418874

[B45] Xuan J. Yu Y. Qing T. Guo L. Shi L. (2013). Next-generation sequencing in the clinic: promises and challenges. Cancer Lett. 340, 284–295. Elsevier Ireland Ltd. 10.1016/j.canlet.2012.11.025 23174106 PMC5739311

[B46] Yu S. L. Zhang H. Ho B. C. Yu C. H. Chang C. C. Hsu Y. C. (2020). FPGS relapse-specific mutations in relapsed childhood acute lymphoblastic leukemia. Sci. Rep. 10 (1), 12074. 10.1038/s41598-020-69059-y 32694622 PMC7374087

[B47] Zhang H. Wang H. Qian X. Gao S. Xia J. Liu J. (2020). Genetic mutational analysis of pediatric acute lymphoblastic leukemia from a single center in China using exon sequencing. BMC Cancer 20 (1), 211. 10.1186/s12885-020-6709-7 32164600 PMC7068927

[B48] Zou P. Zhou M. Wen J. Liao X. Shen Y. Liu H. (2022). The long-term outcome and risk factors for precursor B cell acute lymphoblastic leukemia without specific fusion genes in Chinese children: experiences from multiple centers. Bosn. J. Basic Med. Sci. 22 (2), 238–246. 10.17305/bjbms.2021.5879 34392828 PMC8977091

